# A systematic review of the incidence of hypersensitivity reactions and post-contrast acute kidney injury after ioversol: part 2—intra-arterial administration

**DOI:** 10.1007/s00330-022-08637-2

**Published:** 2022-03-21

**Authors:** Aart J. van der Molen, Ilona A. Dekkers, Ibrahim Bedioune, Elisabeth Darmon-Kern

**Affiliations:** 1 Contrast Media Safety Research Group, Department of Radiology C-2S, Leiden University Medical Center, Albinusdreef 2, NL-2333 ZA Leiden, The Netherlands; 2 Clinical Development Department, Guerbet, Roissy CDG Cedex, France

**Keywords:** Ioversol, Contrast media, Injections, intra-arterial, Acute kidney injury, Drug-related side effects and adverse reactions

## Abstract

**Objectives:**

To evaluate the incidence of adverse drug reactions (ADRs), including hypersensitivity reactions (HSRs) and post-contrast acute kidney injury (PC-AKI), after intra-arterial (IA) administration of ioversol.

**Methods and materials:**

A systematic literature search was performed (1980–2021) and studies documenting IA use of ioversol, and reporting safety outcomes were selected. Key information on study design, patients’ characteristics, indication, dose, and type of safety outcome were extracted.

**Results:**

Twenty-eight studies (including two pediatric studies) with 8373 patients exposed to IA ioversol were selected. Studies were highly heterogenous in terms of design, PC-AKI definition, and studied population. PC-AKI incidence after coronary angiography was 7.5–21.9% in a general population, 4.0-26.4% in diabetic patients, and 5.5–28.9% in patients with chronic kidney disease (CKD). PC-AKI requiring dialysis was rare and reported mainly in patients with severe CKD. No significant differences in PC-AKI rates were shown in studies comparing different iodinated contrast media (ICM). Based on seven studies of ioversol clinical development, the overall ADR incidence was 1.6%, comparable to that reported with other non-ionic ICM. Pediatric data were scarce with only one study reporting on PC-AKI incidence (12%), and one reporting on ADR incidence (0.09%), both after coronary angiography.

**Conclusions:**

After ioversol IA administration, PC-AKI incidence was highly variable between studies, likely reflecting the heterogeneity of the included study populations, and appeared comparable to that reported with other ICM. The rate of other ADRs appears to be low. Well-designed studies are needed for a better comparison with other ICM.

**Key Points:**

*• PC-AKI incidence after IA administration of ioversol appears to be comparable to that of other ICM, despite the high variability between studies.*

*• The need for dialysis after IA administration of ioversol is rare.*

*• No obvious difference was found regarding the safety profile of ioversol between IA and IV administration.*

## Introduction

Ioversol (Optiray®, Guerbet), a non-ionic monomeric low-osmolar iodinated contrast medium (ICM, LOCM), has been used for more than three decades in a variety of X-ray-based modalities involving intravenous (IV) administration such as CT, angiography, and venography or intra-arterial (IA) administration such as coronary, cerebral, or peripheral angiography.

Complications after the use of ICM include hypersensitivity reactions (HSRs), which can be either immediate or non-immediate [1], and post-contrast acute kidney injury (PC-AKI), typified by a deterioration of renal function after intravascular injection of ICM and associated with increased morbidity, mortality, and longer hospital stay especially in patients with chronic kidney disease (CKD) [2–5].

Whether the incidence of adverse drug reactions (ADRs) and specifically HSRs is higher after IA or IV administration of ICM is still debated, and comparative studies are lacking. For instance, a nested case-control analysis of more than 133,000 patients exposed to iopromide showed that HSRs were significantly less frequent after IA administration compared to IV administration [6]. On the other hand, a phase II study showed that ADR incidence was relatively higher with IA administration of both iodixanol and iosimenol compared to IV administration [7].

In contrast with IV and IA administration with second-pass renal exposure (such as right heart, pulmonary, carotid, subclavian, brachial, coronary, mesenteric, iliac, femoral arteries administration), the ICM after IA administration with first-pass renal exposure (such as left heart, thoracic and suprarenal abdominal aorta, and renal arteries administration) reach the renal arteries in a relatively concentrated form, hence increasing PC-AKI risk [8, 9]. PC-AKI incidence may be higher in studies involving ICM IA vs. IV administration. This could be due to the fact that cardiac angiography is performed with catheters, which can dislodge athero-emboli, and the ICM dose in this procedure may be more abrupt and concentrated to the kidneys [10], especially from backflow of ICM from the coronary arteries into the aortic arch or when arch angiography or left ventriculography is part of the examination.

In part 1 of our systematic review [11], it has been shown that ADR/HSR incidence with IV ioversol (0.13–1.80%, depending on the outcome), especially those of severe intensity (0–0.02%), was among the lowest compared to other ICM. The reported PC-AKI incidence was variable (1–42%); nevertheless, ioversol exposure *per se* did not seem to increase PC-AKI incidence.

In this part 2, we sought to analyze the incidence of ADRs, HSRs, and PC-AKI after IA administration of ioversol and to position its safety profile among the different ICM.

## Materials and methods

This systematic literature review was performed according to the Preferred Reporting Items for Systematic Reviews and Meta-Analyses (PRISMA) guidelines [12]. The detailed methodology was previously published [11]. Briefly, MEDLINE (PubMed) and EMBASE (Elsevier) were systematically searched for studies published between January 1980 and May 2021 using keywords evocative of ICM–associated adverse events such as “allergic reaction,” “hypersensitivity,” “anaphylactic,” “nephrotoxicity,” and “kidney injury.”

### Study selection

Clinical studies documenting IA administration of ioversol and other ICM and the presence or absence of ADRs, and/or HSRs, and/or PC-AKI were selected. Reviews, commentaries, letters, or case reports were excluded. Studies with < 5 patients were excluded. Study selection was conducted and reconciled between two independent authors. Publications were first screened based on title and abstract, then a full-text screening was performed. Additional publications were identified by cross-referencing.

### Data extraction and study quality assessment

Key information, such as patient characteristics, type of procedure, number of patients, administered dose, type of safety outcome, and incidence, was extracted. When PC-AKI was the outcome of interest, its definition was also extracted.

Methodological quality of non-randomized studies was assessed as previously described [11] using a modified Newcastle-Ottawa scale (NOS) [13] with a score ranging from 0 to 8, based on eight questions related to patient selection, comparability of cohorts, and outcome assessment. Scores of 7–8 and 5–6 indicated high- and moderate-quality studies, respectively. The revised Cochrane Risk of Bias assessment tool for randomized trials (ROB 2) algorithm was used for randomized controlled trials (RCT) [13, 14]. The heterogeneity between studies reporting PC-AKI incidence was assessed using *I*^2^ statistics.

## Results

### Study selection

**Fig. 1:**
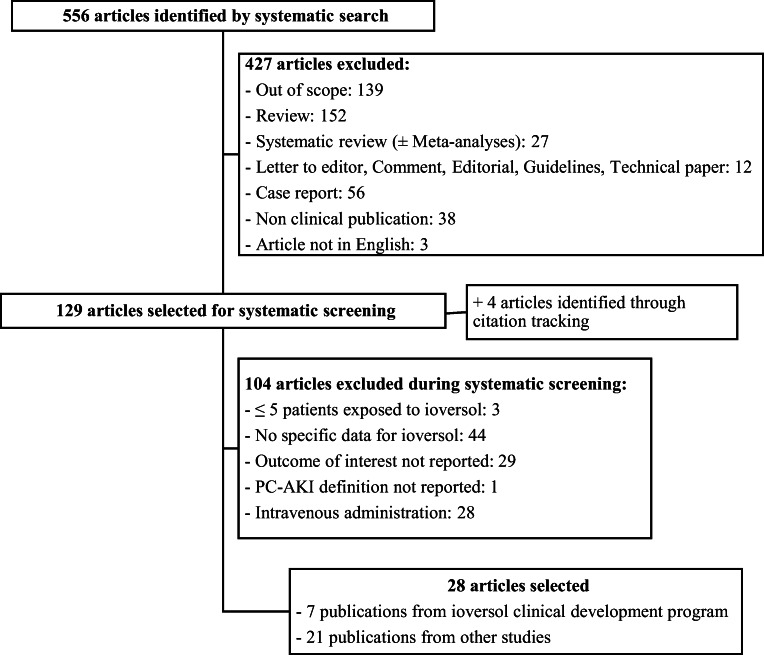
Flow diagram of the search strategy and study selection

The systematic search identified 556 articles, and a full-text screening was performed for 129 articles. Twenty-eight studies were selected [15–42], including four identified through citation tracking and two performed on pediatric patients [24, 28] (Fig. 1). The selected studies included 8373 patients (2412 pediatric) exposed to ioversol.

Multiple ICM were used in 10 studies [16, 23, 27, 30, 33–36, 38, 39] and seven were randomized for ICM allocation [27, 33–36, 38, 39]. In studies where the NOS was applied, three were of high quality and 19 of medium quality. RCT for ICM allocation had a low risk of bias (Table 1).

**Table 1:** Description of all selected studies

Study	Country	Study Design	Indication & Population	Renal exposure	Age & Gender	Contrast Media	Dose	N patients	Quality Score ^a^
Outcome: PC-AKI incidence									
Azzalini 2018 [16]	Italy	R, S	PCI in adult patients	2^nd^	Ioversol 68.0 ± 11.5 years Male: 81% Iopromide 67.9 ± 11.3 years Male: 82% Iomeprol 68.1 ± 11.6 years Male: 81% Iobitridol 68.1 ± 11.1 years Male: 80% Iodixanol 68.5 ± 11.4 years Male: 80%	**Ioversol 350** Iopromide 370 Iomeprol 350 Iobitridol 350 Iodixanol 320	232 ± 113 mL 233 ± 123 mL 228 ± 126 mL 243 ± 129 mL 209 ± 111 mL	272 818 611 460 487	7
El-saadani 2017 [22]	Egypt	RCT^b^, O, S	PCI in adult patients	2^nd^	52.3± 6.6 years Male: 61.7%	Ioversol 350	NR	60	5
Fu 2018 [42]	China	RCT^b^, M	PCI in adult patients	2^nd^	Probucol group 60.3 ± 11.7 years Male: 57% Control group 61.9 ± 12.4 years Male: 59%	Ioversol	Probucol group 148 ± 11 mL Control group 150 ± 11 mL	641	5
Cao 2018 [40]	China	RCT^b^, S	PCI in adult patients	2^nd^	Male: 88% With RIPostC 59 ±13 years Without RIPostC 59 ±11 years	Ioversol	With RIPostC 88 ± 23 mL Without RIPostC 92 ± 20 mL	64	5
Call 2006 [19]	USA	P, S	Coronary angiography or PCI in adult patients	2^nd^	Automated injection 64.9±12.5 years Male: 63.9% Hand injection 64.0±12.6 years Male: 63.0%	Ioversol 350	Automated injection 145.6±107.6 mL Hand injection 204.3±147.1 mL	2175	6
Hernandez 2009 [23]	Spain	P, S, O	Coronary angiography with or without PCI in diabetic patients	2^nd^	Ioversol 70.1±7.9 years Male: 64.4% Iodixanol 69.1±9.0 years Male: 61.9%	**Ioversol 350** Iodixanol 270	195.5±92.1 mL 194.5±80.7 mL	132 118	7
Zaki 2015 [32]	Egypt	P, S	Coronary angiography or PCI in diabetic patients	2^nd^	57.73±7.50 years Male: 64.8%	Ioversol 300	165.9 ±88.9 mL	250	5
Wróbel 2010 [31]	Poland	RCT^b^, S	Coronary angiography and/or PCI in diabetic patients	2^nd^	67±7.8 years Male: 56.9%	Ioversol 350	IV hydration 101.1 ± 36.6 mL Oral hydration 110.4 ± 65.3 mL	102	7
Rudnick 2008 [27]	USA & Canada	RCT, DB, M	Coronary angiography in patients with CKD	2^nd^	Ioversol 72.6 ± 10.2 years Male: 73.8% Iodixanol 71.1 ± 9.9 years Male: 68.2%	**Ioversol 320** Iodixanol 320	129.9 ± 80.8 mL 118.4 ± 83.8 mL	143 156	Low risk of bias
Cho 2010 [20]	USA	RCT^b^, S	Coronary angiography in patients with CKD	2^nd^	78±8 years Male: 50.5%	Ioversol 320	IV fluid 122.6 mL IV bicarbonate & fluid 136.3 mL Oral fluid 118.6 mL Oral bicarbonate & fluid 136.5 mL	91	5
Baskurt 2009 [18]	Turkey	RCT^b^, S	Coronary angiography in patients with moderate CKD	2^nd^	67.4±9.8 years	Ioversol	IV hydration 113.5±37.7 mL IV hydration + NAC 115.6±35.2 mL IV hydration + NAC+Theophilline 130.7±44.5 mL	217	5
Azzalini 2019 [17]	Italy	R, S	Coronary angiography with PCI in patients with severe CKD	2^nd^	76 (71-81) years Male: 76%	Ioversol	85 (50-140) mL	111	5
Abaci 2015 [15]	Turkey	RCT^b^, S	Coronary or peripheral angiography in patients with CKD	2^nd^	Control 67.7±8.9 years Male: 73.4% Rosuvastatin 67.5±8.9 years Male: 64%	Ioversol	Control 117.7±56.8 mL Rosuvastatin 139.2±77.4 mL	208	5
Komenda 2007 [25]	Canada	R^c^, S	Coronary or peripheral vessel angiography or angioplasty and stenting in patients with CKD	1^st^	64±13.8 years Male: 48%	Ioversol 320	NR	31	5
Cury 2018 [21]	Brazil	P, S	Peripheral angiography in patients with critical limb ischemia	2^nd^	70.5±10.7 years Male: 57%	Ioversol 320	148.5±79.4 mL	107	5
Sharma 2013 [29]	USA	R, S	Cerebral angiography in patients undergoing endovascular treatment of acute ischemic stroke	2^nd^	65±14 years Male: 48%	Ioversol 320	150 mL	194	5
Krol 2007 [26]	Canada	R, S	Cerebral angiography in patients with acute stroke syndrome	2^nd^	68.2±14.1 years Male: 62%	Ioversol 320	75-100 mL	224	5
Xu 2017 [41]	China	P, S	Angiography in adult patients	2^nd^	52.1 ± 14.5 years Male: 77%	Ioversol 320	PC-AKI 50±24 mL No PC-AKI 62±10 mL	213	5
Hirsch 2007 [24]	USA	P, S	Coronary angiography in pediatric patients with congenital heart diseases	2^nd^	No PC-AKI 6.6±3.2 years Male: 58% PC-AKI 7.3±3.1 years Male: 55%	Ioversol 350	No PC-AKI 3.4±0.2 mL/kg PC-AKI 4.2±0.6 mL/kg	91	5
Outcome: HSRs incidence									
Sohn 2019 [30]	Korea	P, S	Coronary angiography with or without PCI in adult patients	2^nd^	62.9 ± 10.3 years Male: 71.0%	**Ioversol 320** Iodixanol	NR	416 298	5
Outcome: ADRs incidence									
Cutcliff 1989 [33]	USA	DB, RCT, S	Peripheral and visceral arteriography in adult patients	2^nd^	19-85 years Male: 70%	**Ioversol 320** Iohexol 300	Peripheral procedures 100 mL 96 mL Visceral procedures 87 mL 135 mL	30 30	Low risk of bias
Grassi 1989 [34]	USA	DB, RCT, S	Peripheral and visceral arteriography in adult patients	2^nd^	NR	**Ioversol 320** Diatrizoate 282	Peripheral procedures 159 (41-275) mL 165 (39-247) mL Visceral procedures 178 (76-282) mL 162 (60-334) mL	30 30	Low risk of bias
Ringel 1989 [38]	Germany	DB, RCT, S	Cerebral angiography in adult patients	2^nd^	Ioversol 57 (31-81) years Male: 67% Iopamidol 59 (24-77) years Male: 70%	**Ioversol 320** Iopamidol 300	29 (8-110) mL 23 (11-75) mL	30 30	Low risk of bias
McGaughey 1991 [36]	USA & Germany	DB, RCT, M	Coronary arteriography in adult patients	2^nd^	Ioversol 61 (33-84) years Male: 71% Iohexol 56 (36-78) years Male: 78%	**Ioversol 350** Iohexol 350	123 (61-242) mL 125 (73-230) mL	80 80	Low risk of bias
Hirshfeld 1989 [35]	USA	DB, RCT, S	Coronary angiography in adult patients	2^nd^	Ioversol 59±11 years Male: 70% Iopamidol 59±11 years Male: 87% Diatrizoate 59±12 years Male: 63%	**Ioversol 320** Iopamidol 370 Diatrizoate 370	141±42 mL 131±41 mL 141±41 mL	60 30 30	Low risk of bias
Reagan 1988 [39]	USA	DB, RCT, S	Coronary angiography in adult patients	2^nd^	NR	**Ioversol 320** Diatrizoate 370	NR	40 40	Low risk of bias
Miller 1989 [37]	USA	OL, S	Intra-arterial digital subtraction angiography in adult patients	2^nd^	19-84 years Male: 45%	Ioversol 160	110 mL	40	5
Senthilnathan 2009 [28]	USA	P, S	Coronary angiography in pediatric patients with congenital heart diseases	2^nd^	<1 year: 31% 1-10 years: 48% 11-17 years: 21% Gender: NR	Ioversol 350	3.9 (2-6) mL/kg ^d^	2321	5

*P* Prospective; *R* Retrospective; *RCT* Randomized Controlled Trial; *S* single-center; *M* Multicenter; *DB* double blind; *O* Open label; *PCI* percutaneous coronary intervention; *CKD* chronic kidney disease; *RIPostC* remote ischemic postconditioning; *NR* Not reported

^a^Quality score according to Newcastle-Ottawa Scale (NOS) except for one RCT (Rudnick 2008) assessed with revised Cochrane Risk of Bias assessment tool for randomized trials (ROB 2) algorithm

^b^Randomization for prophylactic treatment allocation

^c^case series

^d^median (interquartile range)

PC-AKI incidence was the main outcome studied (19 studies), while the incidence of ADRs or HSRs was studied in nine studies [28, 30, 33–39]. Coronary angiography was the main type of procedure (19 studies) [15–20, 22–25, 27, 28, 30–32, 35, 36, 39] followed by peripheral (five studies) [15, 21, 25, 33, 34] and cerebral angiography (three studies) [26, 29, 38] (Table 1).

Used ioversol dose was reported in 25 studies. The mean dose was variable in studies with adults and ranged between 29 and 232 mL (101–196 mL in diabetic patients [23, 31, 32] and 85–139 mL in CKD patients who underwent coronary angiography [15, 17, 18, 20, 27]). The mean or median dose in pediatric patients who underwent coronary angiography was ≈ 4 mL/kg [24, 28] (Table 1).

### Post-contrast acute kidney injury

Almost all studies involved IA administration with second-pass renal exposure. A high heterogeneity between studies was observed (*I*^2^ = 92.04%, *p* < 0.0001), and the overall PC-AKI incidence was highly variable (1.5–35.5%), depending on the PC-AKI definition and studied population (Table 2). Prophylactic measures included IV hydration [15–20, 23, 24, 27, 31, 42], anti-hyperlipidemic drugs [15, 22, 42] and N-acetylcysteine administration [18, 19, 23, 27], and bicarbonate infusion [19, 20].

**Table 2:** Incidence of PC-AKI after intra-arterial administration of ioversol

Study	Contrast Media	N Patients	PC-AKI Definition	Incidence (%)				
Azzalini 2018 [16]			AKIN definition:	Overall	St 1	St 2	St 3	Dialysis
	**Ioversol**	272	Stage 1: sCr rise ≥0.3 mg/dL or ≥50-100%;	**13.0%**	**10.3%**	**0.8%**	**1.9%**	**0%**
	Iopromide	818	Stage 2: sCr rise >100-200%;	11.5%	8.7%	1.6%	1.3%	0.5%
	Iomeprol	611	Stage 3: sCr rise >200% or ≥4.0 mg/dL with an acute increase of ≥0.5 mg/dL.	10.2%	9.2%	0.7%	0.3%	0.3%
	Iobitridol	460		13.9%	11.7%	1.3%	0.9%	0%
	Iodixanol	487		11.3%	10%	0.9%	0.4%	0.4%
El-saadani 2017 [22]	Ioversol	60	sCr rise ≥25% within 48h	11.7%				
Fu 2018 [42]	Ioversol	641	sCr rise ≥25% within 72h	7.5%				
Cao 2018 [40]	Ioversol	64	sCr rise ≥25% within 72h	21.9%				
Call 2006 [19]	Ioversol	2175	sCr rise >0.5 mg/dL or >25% within 7 days	Automated injection: 13.3% ^a^ Hand injection: 19.3%				
Hernandez 2009 [23]	**Ioversol**	132	sCr rise >0.5 mg/dL or >25% at 72h post procedure	8.3% ^a^				
	Iodixanol	118		2.5%				
Zaki 2015 [32]	Ioversol	250	sCr rise ≥0.5 mg/dL within 48–72h	23.2%				
Wróbel 2010 [31]	Ioversol	102	sCr rise ≥0.5 mg/dL or ≥25% at 72h post procedure	IV hydration: 5.8% Oral hydration: 4%				
Rudnick 2008 [27]	**Ioversol**	143	sCr rise ≥0.5 mg/dL within 72h	23.8%				
	Iodixanol	156		21.8%				
Cho 2010 [20]	Ioversol	91	sCr rise >0.5 mg/dL or >25% at 72 hours post procedure	11%				
Baskurt 2009 [18]	Ioversol	217	sCr rise >0.5 mg/dL within 48h	5.5%				
Azzalini 2019 [17]	Ioversol	111	AKIN definition:	Overall: 15.5% ^b^				
			Stage 1: sCr rise ≥0.3 mg/dL or ≥50-100%;	9.7%				
			Stage 2: sCr rise >100-200%;	0%				
			Stage 3: sCr rise >200% or ≥4.0 mg/dL with an acute increase of ≥0.5 mg/dL.	5.8%				
Abaci 2015 [15]	Ioversol	208	sCr rise >0.5 mg/dL or ≥25% within 48–72h	7.2%				
Komenda 2007 [25]	Ioversol	31	sCr rise > 25%	9.1%				
Cury 2018 [21]	Ioversol	107	sCr rise ≥25% within 5 days	35.5%				
Sharma 2013 [29]	Ioversol	194	sCr rise >0.3 mg/dL or >50% within 48h	1.5%				
Krol 2007 [26]	Ioversol	224	sCr rise ≥ 25% within 5 days	3%				
Xu 2017 [41]	Ioversol	213	sCr rise >0.3 mg/dL or >50% within 48–72h	8%				
Hirsch 2007 [24]	Ioversol	91	sCr rise ≥ 50%	12%				

*PCI* AKIN: Acute Kidney Injury Network; *sCr* Serum creatinine; *St* Stage

^a^Statistically significant difference

^b^incidence reported for patients who underwent conventional

#### Post-contrast acute kidney injury in general population undergoing PCI or coronary angiography

Five studies included a general population of patients who mainly underwent percutaneous coronary intervention (PCI), and PC-AKI incidence was 7.5–21.9% [16, 19, 22, 40, 42].

In Azzalini et al [16], PC-AKI incidence with ioversol (13%) was not statistically different from that reported with other ICM (10.2–13.9%). The incidence of stage 3 PC-AKI with ioversol was 1.9% compared to 0.3–1.3% with other ICM (no statistical difference). The risk of PC-AKI requiring dialysis was nil in the ioversol group. Propensity score adjustment for multiple treatments showed that all LOCM used in this study had similar adjusted risk of PC-AKI compared to iodixanol.

In El-Saadani et al [22], patients were randomized to three groups (no load, low and high load of atorvastatin). PC-AKI was reported in seven patients (11.7%, mainly in the no-load group) and none needed dialysis.

In Fu et al [42], patients were randomized to receive or not probucol (anti-hyperlipidemic drug with antioxidant properties). PC-AKI incidence was 7.5%, and probucol + hydration was more effective in decreasing PC-AKI incidence (4% vs. 11% in the hydration group, *p* = 0.01). One patient in the hydration group required temporary dialysis.

In Cao et al [40], patients underwent PCI with or without upper arm remote ischemic postconditioning (RIPostC). PC-AKI incidence was 21.9%, and RIPostC was more protective against PC-AKI incidence (10% vs. 31% in the control group, *p* = 0.04).

Call et al [19] included patients who underwent hand or automated injection of ioversol. PC-AKI incidence was significantly lower in the automated injection group (13.3% vs. 19.3% for hand injection).

### Post-contrast acute kidney injury in diabetic patients undergoing coronary angiography or PCI

PC-AKI incidence in diabetic patients who underwent coronary angiography and/or PCI was reported in five studies (4.0–26.4%) [19, 23, 27, 31, 32].

In Hernandez et al [23], where 70% of patients had an estimated glomerular filtration rate (eGFR) ≥ 60 mL/min/1.73 m^2^, PC-AKI incidence was 8.3% with ioversol and 2.5% with iodixanol (*p* = 0.047). None required dialysis. The type of ICM was found as an independent predictor of PC-AKI while ICM volume was not.

In Zaki et al [32], 78.4% of patients had an eGFR > 90 mL/min and no prophylactic measures were undertaken. PC-AKI incidence was 23.2% and none required dialysis. PC-AKI incidence in patients who underwent coronary angiography was significantly lower than in those who underwent PCI (11.4% vs. 43.5%, respectively).

In Wróbel et al [31], PC-AKI incidence was comparable between patients who had oral hydration for PC-AKI prevention (4%) and those IV hydrated (5.8%). None required dialysis.

In Call et al [19], PC-AKI incidence in diabetic patients was 18.7% in the automated injection group and 23.4% in the hand injection group (*p* = 0.26). In Rudnick et al [27], PC-AKI incidence in diabetic patients with CKD was 26.4% with ioversol and 21.9% with iodixanol (*p* = 0.57).

#### Post-contrast acute kidney injury in CKD patients undergoing coronary angiography or PCI

PC-AKI incidence in CKD patients who underwent coronary angiography and/or PCI was reported in five studies (5.5–28.9%) [15, 17–20, 23, 27].

Rudnick et al [27] is a double-blind study where patients were randomly administered ioversol or iodixanol. Overall, PC-AKI incidence with ioversol was 23.8% compared to 21.8% with iodixanol (*p* = 0.78).

In Cho et al [20], patients were randomized to four prophylactic groups (Table 1). Overall PC-AKI incidence was 11%, and no significant difference was observed between the four groups.

In Baskurt et al [18], patients were randomized to three prophylactic groups: IV hydration with normal saline alone (group 1) or supplemented with N-acetylcysteine (group 2) or with N-acetylcysteine + theophylline (group 3). Overall PC-AKI incidence was 5.5% (12 patients): five patients in group 1 (6.9%) and seven in group 2 (9.6%). None needed dialysis. In Abaci et al [15], patients who underwent coronary or peripheral angiography were assigned to receive rosuvastatin or not (control group). The overall PC-AKI incidence was 7.2%, none required dialysis, and no significant difference was observed between the groups. The incidence for each indication was not provided.

In Azzalini et al [17], severe CKD patients underwent an ultra-low contrast volume PCI (ULC-PCI, *n* = 8, mean of 8.8 mL) or a conventional PCI (*n* = 103, 90 mL). No cases of PC-AKI were reported in the ULC-PCI group. PC-AKI incidence in the conventional PCI group was 15.5%. Dialysis was needed in five patients (4.9%). The difference in PC-AKI incidence between the two groups was not statistically significant.

In Hernandez et al [23], PC-AKI incidence in diabetic patients with CKD was 17.1% with ioversol (vs. 4.9% with iodixanol, *p* = 0.082). In Call et al [19], PC-AKI incidence in CKD patients was 21.6–28.9% depending on the injection group.

#### Post-contrast acute kidney injury in pediatric patients undergoing coronary angiography

In Hirsch et al [24], PC-AKI in pediatric patients with congenital heart disease undergoing coronary angiography was reported in 11 patients (12%) (sCr change ≥ 50% at 6 h in five patients and at 24 h post-procedure in six patients).

#### Post-contrast acute kidney injury in patients undergoing direct renal stenting

In Komenda et al [25], PC-AKI incidence in CKD patients who underwent stenting of renal artery stenosis (77%) or coronary angiography (23%) was 9.1% and none required dialysis.

#### Post-contrast acute kidney injury in other indications

Four studies investigated PC-AKI incidence in other angiographic procedures [21, 26, 29, 41].

Cury et al [21], included patients who underwent lower limb angiography for critical limb ischemia. All patients were IV hydrated with a normal saline solution before and after the procedure. Overall, 69% of the patients were diabetic and 21.4% had a stage 3 CKD. PC-AKI incidence was 35.5% and none required dialysis.

Sharma et al [29] included patients who underwent endovascular treatment for acute ischemic stroke. Overall, 25% of patients were diabetic and 16% had CKD. PC-AKI was reported in three patients (1.5%) including one who had CKD.

In Krol et al [26], patients undergoing cerebral angiography had a PC-AKI incidence of 3% and none required dialysis.

In Xu et al [41], PC-AKI incidence in patients undergoing angiography was 8% using a sCr–based definition and 24% with a serum cystatin C (sCys C)–based definition. None required dialysis.

### Other safety outcomes

Nine studies reported other safety outcomes [28, 30, 33–39]. In seven small studies of ioversol clinical development (310 patients) [33–39], ADRs were reported in five patients (1.6%) and consisted of urticaria, nausea, angina (one patient each), angina and chills in one patient (doubtfully related to contrast), dizziness and blurred vision in another patient. Incidence was comparable to that reported with other non-ionic LOCM (Table 3).

**Table 3:** Incidence of ADRs after intra-arterial administration of ioversol

Study	Contrast Media	N Patients	Incidence (%)
Cutcliff 1989 [33]	**Ioversol**	30	0%
	Iohexol	30	3.3%
Grassi 1989 [34]	**Ioversol**	30	3.3%
	Diatrizoate	30	16.7%
Ringel 1989 [38]	**Ioversol**	30	3.3%
	Iopamidol	30	6.7%
McGaughey 1991 [36]	**Ioversol**	80	0%
	Iohexol	80	2.5%
Hirshfeld 1989 [35]	**Ioversol**	60	1.7%
	Iopamidol	30	0%
	Diatrizoate	30	20%
Reagan 1988 [39]	**Ioversol**	40	5%
	Diatrizoate	40	7.5%
Miller 1989 [37]	Ioversol	40	0%
Senthilnathan 2009 [28]	Ioversol	2321	0.09%

In Sohn et al [30], the incidence of immediate HSRs in patients who underwent coronary angiography was 2.7% and 5.3% and that of delayed HSRs was 12.5% and 18.8% (*p* = 0.022) with ioversol and iodixanol, respectively. Two severe HSRs were reported and PC-AKI incidence was 0.7%, but no difference between the two ICM was reported.

Senthilnathan et al [28] included pediatric patients requiring coronary angiography with or without intervention. ADRs possibly related to ioversol were reported in two patients (0.09%): dizziness, slurred speech, and amnesia in a 13-year-old patient, and PC-AKI in a 1-day-old patient.

## Discussion

In this systematic review investigating the incidence of ADRs, HSRs, and PC-AKI after IA administration of ioversol, most of the selected studies focused on PC-AKI incidence after IA administration with second-pass renal exposure.

PC-AKI incidence after IA administration of ioversol was highly variable and ranged between 1.5 and 35.5%. This could be due to several factors such as the study design, clinical practice according to different countries, studied populations (general population, CKD or diabetic patients), indication (coronary angiography with or without intervention, cerebral or peripheral angiography), and, finally, the variety of definitions used in these studies.

In patients who underwent coronary angiography with or without PCI with ioversol, PC-AKI incidence was 7.5–21.9% in a general population, 4.0–26.4% in diabetic patients, and 5.5–28.9% in patients with CKD. PC-AKI incidence was comparable in three of four studies using multiple ICM. The fourth study [23] had a major limitation as patients were not treated within the same period. One comparative study using ioversol, iohexol, and iopamidol showed a similar rate of PC-AKI readmission within 30 days (i.e., 0.1%) [43]. A recent review [5] reported PC-AKI incidences of 2.7–15% in patients undergoing coronary angiography with or without PCI and 3.2–20.6%. in patients undergoing PCI, similar to the incidences reported with ioversol.

A similar high variability in PC-AKI incidence was reported after IV administration of ioversol (1–42%) [11]. This heterogeneity was due to the same reasons as those described above for IA administration, and is therefore limiting the possibility to draw conclusions regarding the two administration routes. Nevertheless, as most selected studies for this review involved second-pass renal exposure, differences in PC-AKI incidence with IV administration are not expected. In patients who underwent IV or IA iobitridol administration, PC-AKI was more frequent in those who underwent cardiac catheterization angiography (13.2%) compared to coronary CT angiography (5.6%) [44]. Conversely, other studies with patients who underwent IA diagnostic or interventional procedures and IV contrast-enhanced CT showed no difference in PC-AKI incidence [45–49]. Overall, this is supporting the idea that the risk of PC-AKI is similar between IV and IA administration with second-pass renal exposure.

It is clear that renal impairment is the most important risk factor for PC-AKI [10, 50]. In the past, diabetes *per se* was not considered as an independent risk factor for PC-AKI [8, 51, 52]. In a recent meta-analysis of 1.1 million contrast-exposed patients, diabetes mellitus was significantly associated with PC-AKI in CKD patients but not in patients with normal renal function [53]. These results suggest that appropriate PC-AKI prophylactic measures should be taken in diabetic patients with renal impairment (e.g., IV bicarbonate and/or saline hydration, withholding metformin) [54].

Few studies have specifically examined PC-AKI in pediatric populations. One case of PC-AKI was reported in a 1-day-old patient by Senthilnathan et al [28]. However, the authors identified other factors such as gentamicin and diuretics administration that could have contributed to the renal dysfunction in addition to the high ioversol dose, which may reflect the complexity of the procedure.

A low incidence of PC-AKI after cerebral angiography (1.5–3%) was reported with ioversol. The proportion of patients with CKD was low (0.9%) in one study [26] and represented 16% of all patients in the second study, with a PC-AKI incidence in these CKD patients of 3.2% [29]. Other studies performed on CKD patients showed PC-AKI incidences between 0.54 and 20.3%, depending on the ICM used (iodixanol, iopamidol, iohexol, or iomeprol) [55–57]. Thus, it could be reasonably concluded that PC-AKI incidence is low in patients receiving ioversol for cerebral angiography.

PC-AKI incidence was higher (35.5%) in patients who underwent lower limb angiography for critical limb ischemia with ioversol [21]. Likewise, a systematic review with more than 11,300 patients highlighted high incidences of PC-AKI for this type of procedures (range 0–45% and median of 10%) [58].

PC-AKI requiring dialysis was rare and mainly reported in patients with severe CKD [17], consistent with what has been reported with other ICM (0.3–1.5%) [16, 43, 59–61].

A meta-analysis showed that the risk of PC-AKI after IA administration was not significantly lower with iodixanol, overall and in CKD patients [62]. Another showed significant PC-AKI risk reduction using the sCr increase definition “≥ 0.5 mg/dL” but not “≥ 25%” [63]. Moreover, both studies showed reduced PC-AKI risk with iodixanol as compared to iohexol. However, it is not clear whether the IOCM iodixanol is different from other LOCM regarding clinical outcomes such as the need for hemodialysis, progression of CKD, rehospitalization, or mortality.

Only few studies investigated HSR incidence after IA administration of ioversol. HSR incidences after IA administration were higher than those reported in studies with IV administration of ioversol (0.2–1.8%) [11]. In contrast, a study with > 152,000 patients from four pooled observational studies with iopromide showed that HSR incidence was significantly more frequent after IV administration (0.7% vs. 0.2% with IA administration) [6]. The median ADR incidence calculated from ioversol clinical development studies was 1.7% for IA procedures, comparable to that reported with IV procedures [11]. In pediatric patients, ADR incidence after IA administration of ioversol (0.09%) [28] was lower than reported with IV administration (0.38%) [64]. However, due to the limited number of studies, it is difficult to infer whether ADR and HSR incidences are different between IA and IV administration.

This study comes with some limitations. Only one study reported the incidence of HSRs after IA administration of ioversol. Nevertheless, the incidence of ADRs reported in several studies was low and comparable to that of other LOCM. Regarding PC-AKI, only three studies used other ICMs in addition to ioversol, of which only one was a RCT. Therefore, we were unable to compare the incidence of PC-AKI between ioversol and other ICM within selected studies. PC-AKI incidences were highly variable due, inter alia, to various definitions used in the selected studies and the analyzed patients’ populations. However, analysis of the literature did not highlight differences between ioversol and other LOCM. Data on pediatric populations are limited as only two studies were identified with relevant data.

In conclusion, PC-AKI incidence after IA administration of ioversol was highly variable between studies and reflects the heterogeneity of the included study populations. Nevertheless, PC-AKI incidence appears comparable to what has been reported in the literature with other ICM and PC-AKI requiring dialysis was mainly reported in patients with severe CKD. The rate of other outcomes appears to be low, therefore highlighting the good safety profile of ioversol. Well-designed studies are needed for a better comparison with other ICM, for evaluation of other safety endpoints, and in pediatric populations.

## Abbreviations

ADR: Adverse drug reaction
CKD: Chronic kidney disease
eGFR: Estimated glomerular filtration rate
HSR: Hypersensitivity reaction
IA: Intra-arterial
ICM: Iodinated contrast medium
IV: Intravenous
LOCM: Low-osmolar contrast medium
NOS: Newcastle-Ottawa scale
PC-AKI: Post-contrast acute kidney injury
PCI: Percutaneous coronary intervention
PRISMA: Preferred Reporting Items for Systematic Reviews and Meta-Analyses
RCT: Randomized controlled trial
RIPostC: Remote ischemic postconditioning
ROB 2: Revised Cochrane Risk of Bias assessment tool for randomized trials
sCr: Serum creatinine
sCysC: Serum cystatin C

## Declarations

### Guarantor

The scientific guarantor of this publication is Aart J. van der Molen.

### Statistics and Biometry

No complex statistical methods were necessary for this paper.

### Study subjects or cohorts overlap

Studies with duplicate data was excluded from this systematic review.

### Methodology

• Multicenter study
